# Prognostic Values of TIM-3 Expression in Patients With Solid Tumors: A Meta-Analysis and Database Evaluation

**DOI:** 10.3389/fonc.2020.01288

**Published:** 2020-08-04

**Authors:** Shuang Qin, Bing Dong, Ming Yi, Qian Chu, Kongming Wu

**Affiliations:** ^1^Department of Oncology, Tongji Hospital of Tongji Medical College, Huazhong University of Science and Technology, Wuhan, China; ^2^Department of Radiation Oncology, Hubei Cancer Hospital, Tongji Medical College, Huazhong University of Science and Technology, Wuhan, China; ^3^Department of Molecular Pathology, The Affiliated Cancer Hospital of Zhengzhou University & Henan Cancer Hospital, Zhengzhou, China

**Keywords:** TIM-3, solid tumor, prognosis, overall survival, meta-analysis

## Abstract

**Background:** T cell immunoglobulin and mucin-domain containing molecule-3 (TIM-3), a novel emerging immune checkpoint molecule, was reported to express both on various kinds of immune cells and tumor cells. Many previous studies have investigated the prognostic significance of TIM-3 in cancer. However, the sample number from single study was limited and results remained controversial.

**Methods:** We searched PubMed, Web of Science, and Embase databases for publications concerning TIM-3 expression in solid cancers up to March 2020. The correlations between TIM-3 and survival as well as clinical-pathological features were analyzed. Pooled hazard ratios (HRs), odds ratios (ORs), and 95% confidence interval (CI) were estimated by either fixed or random effects models.

**Results:** A total of 3,072 patients were included in our meta-analysis. The result suggested that TIM-3 protein overexpression was relevant to poor overall survival (HR = 1.73, 95% CI = 1.39–2.15, *P* < 0.001). Moreover, TIM-3 was shown to be connected with lymph node metastasis (N+ vs. N-, OR = 1.59, 95% CI = 1.10–2.29, *P* = 0.013), tumor grade (G2-3 vs. G1, OR = 1.68, 95% CI = 1.21–2.34, *P* = 0.002), as well as PD-1 expression (PD-1^high^ vs. PD-1^low^, OR = 3.26, 95% CI = 2.20–4.82, *P* < 0.001). In database test, significant correlations between high TIM-3 mRNA expression and poor overall survival for patients with non-small cell lung cancer and gastric cancer were observed (HR = 1.46, 95% CI = 1.23–1.72, *P* < 0.001; HR = 1.41, 95% CI = 1.12–1.77, *P* = 0.0038).

**Conclusion:** Our meta-analysis highlights that TIM-3 has the potential to serve as a prognostic marker and a valuable therapeutic target in solid tumors.

## Introduction

Cancer is the leading cause of death all over the world (1). Although tremendous advances with regard to diagnostic technology and therapeutic approaches have been achieved in recent decades, the outcome of most cancers is still far from satisfactory, especially in late stages. Therefore, identifying novel biomarkers which can better predict cancer progression and prognosis is of great value.

T cell immunoglobulin and mucin-domain containing molecule-3 (TIM-3), also called hepatitis A virus cellular receptor 2 (HAVCR2), is a member of the TIM gene family (2). Kuchroo's group firstly explored the function of TIM-3, and described it as a cell surface molecule which can distinguish T helper 1 (Th1) cells and Th2 cells (3). This gene family is named as TIM on account of these proteins are expressed by T cells and encompass an immunoglobulin variable region (IgV)-like domain and a mucin-like domain (4).

In addition to its well-known expression on T cells (3, 5, 6), TIM-3 has also expressed on other cells, such as dendritic cells (DCs), monocytes, and natural killer (NK) cells (7). Recently, a growing body of evidence has shown that the expression of TIM-3 is upregulated in a series of cancers, like colorectal cancer (CRC) (8), gastric cancer (GC) (9), hepatocellular carcinoma (HCC) (10), non-small cell lung cancer (NSCLC) (11), clear cell renal cell carcinomas (RCC) (12), bladder urothelial carcinoma (BUC) (13), prostate cancer (14), and leukemic stem cells (15). There are at least 4 ligands binding to the IgV domain of TIM-3: galectin-9, phosphatidylserine, high-mobility group protein B1 (HMGB1), and carcinoembryonic antigen cell adhesion molecule 1 (Ceacam-1) (16, 17). An in-depth study conducted by Li et al. illustrated that TIM-3 was expressed on tumor-infiltrating CD4+ and CD8+ T cells in HBV-associated HCC. TIM-3+ T cells expressed surface markers for senescence and exhibited decreased proliferative ability. They further demonstrated that the blockade of TIM-3/galectin-9 signaling by anti-TIM-3 monoclonal antibody (mAb) can restore the function of effector T cells, increasing the production of interleukin 2 (IL-2) and IFN-γ (10). Nowadays, several anti-TIM-3 mAbs were currently in clinical trials, including TSR-022, MBG453, and Sym023 (18). Elevated TIM-3 level was related to patients clinical-pathological and prognosis. It was reported that high TIM-3 expression was positively correlated with tumor size, TNM staging and distant metastasis in CRC (8). In the meanwhile, elevated TIM-3 was detected in prostate cancer patients with higher clinical stage (19). In some types of cancers, high expression of TIM-3 has been associated with poor prognosis, while in others the opposite relationship has been observed. For example, Yang et al. reported that high TIM-3 expression was correlated with poor survival in BUC, and it was also related to another immune checkpoint molecule programmed cell death protein-1 (PD-1) (13). On the contrary, Wu et al. found that low expression of TIM-3 in prostate cancer was an independent prognostic factor of bad prognosis (14). Thus, the prognostic value of TIM-3 to predict the outcome in various cancers is controversial. In this setting, we conducted this meta-analysis in order to gain a comprehensive understanding of the prognostic effect of TIM-3 on patients with solid cancers.

## Methods

### Search Strategy

Our meta-analysis was performed in accordance with the Preferred Reporting Items for Systematic Reviews and Meta-Analyses (PRISMA) statement (20). Relevant studies which were published before March 2020, were searched through electronic platforms of PubMed, Web of Science, and Embase databases by two authors (Shuang Qin and Bing Dong) independently. The following keywords were used for our search: “TIM-3,” “TIM3,” “T cell immunoglobulin and mucin-domain containing molecule-3,” “HAVCR2,” “hepatitis A virus cellular receptor 2,” “cancer,” “carcinoma,” “tumor,” “prognostic,” “survival,” “prognosis,” “recurrence,” “outcome,” and “mortality.” Furthermore, reference lists were screened manually to identify potentially related studies. The researches included were restricted to study on human published in English. Any disagreements were settled by discussion and consensus between the two authors.

### Inclusion and Exclusion Criteria

Publications were included in our meta-analysis if they met the following criteria: [1] A definite source of the study was reported; [2] The sample size was more than 30; [3] The expressions of TIM-3 in tumor cells and/or tumor infiltrating lymphocytes (TILs) were measured by immunohistochemical (IHC) staining; [4] The relationships between TIM-3 and overall survival (OS)/disease-free survival (DFS)/progression-free survival (PFS) were described; [5] Hazard ratio (HR) and 95% confidence interval (CI) were reported, or with essential data to calculate it; [6] Studies were published in English. Studies were excluded if they were: (a) duplicate studies; (b) case reports, review articles, letters, conference abstracts, animal studies, or meta-analysis; (c) unpublished data.

### Data Extraction

The following information was recorded: first author, year of publication, patient source, sample size, cancer type, detection of method, expression location, cut-off value, median follow-up, outcome, method to estimate HR (univariate and multivariate analysis), and HR ratio. Multivariate Cox analysis should be given priority if available, or univariate hazard analysis was instead (21). For studies that presented only Kaplan-Meier curves, Engauge Digitizer (version 4.1) was used to extract the survival data and estimate HRs with 95% CIs calculated by Tierney's method (22). Two investigators reviewed the eligible articles independently, then compared their datasets. Any discrepancy between the reviewers was resolved by discussion.

### Quality Assessment

To control the quality of each study, all included articles were scored according to the Newcastle-Ottawa Quality Assessment Scale (NOS) by two investigators independently (23). The NOS criteria was scored on three domains: selection of participants, comparability, and clinical outcome. The score range of NOS was from 0 to 9 (9 as the best), and study with score ≥6 was considered as a high-quality. The mean value of all included studies was a score of 7.6 (ranging from 6 to 9), indicating high quality and good methodology.

### TIM-3 mRNA Expression Profile and Prognosis

An online analysis tool Kaplan-Meier plotter was used to evaluate the effect of TIM-3 mRNA expression levels on OS of NSCLC (24), GC (25), and breast cancer (26) (http://kmplot.com/analysis/). The affymetrix probe ID for TIM-3 was 1554285_at. The follow-up time threshold was set as 120 months. The Kaplan-Meier survival curves downloaded from the website were resized in Adobe Illustrator CS6.

### Statistical Methods

Meta-analysis was performed by STATA software package (version 12.0) (Stata Corp LP, College Station, TX, USA). Cut-off value of TIM-3 extracted from the articles has divided patients into high and low groups. HR and relative 95% CI obtained from the individual study were pooled into a summary HRs to assess the impact of TIM-3 on survival outcome (OS, PFS, and DFS). In addition, odds ratios (ORs) and corresponding 95% CIs were used to measure the relationship between TIM-3 expression and the clinicopathological features. HR or OR higher than 1 indicated a worse prognosis or a significant correlation between TIM-3 and clinical-pathological parameters, respectively. If 95% CI did not include the value 1, the pooled result was considered statistically significant. Heterogeneity among researches were tested using the chi-square-based Q-test and *I*^2^ test. P_H_ value < 0.05 or *I*^2^ > 50% was defined as a significant heterogeneity, then a random effects model was applied to calculate the pooled effect. Otherwise, a fixed effects model was applied. Subgroup analyses were conducted to explore the source of heterogeneity. Begg's and Egger's tests were used to depict the publication bias of all enrolled studies (*P* > 0.05 indicating no publication bias) (27). Additionally, sensitivity analysis was utilized to check the stability of the pooled results and to identify possible explanations for the observed heterogeneity. All *P*-values were two-sided.

## Results

### Study Selection

**Figure 1:**
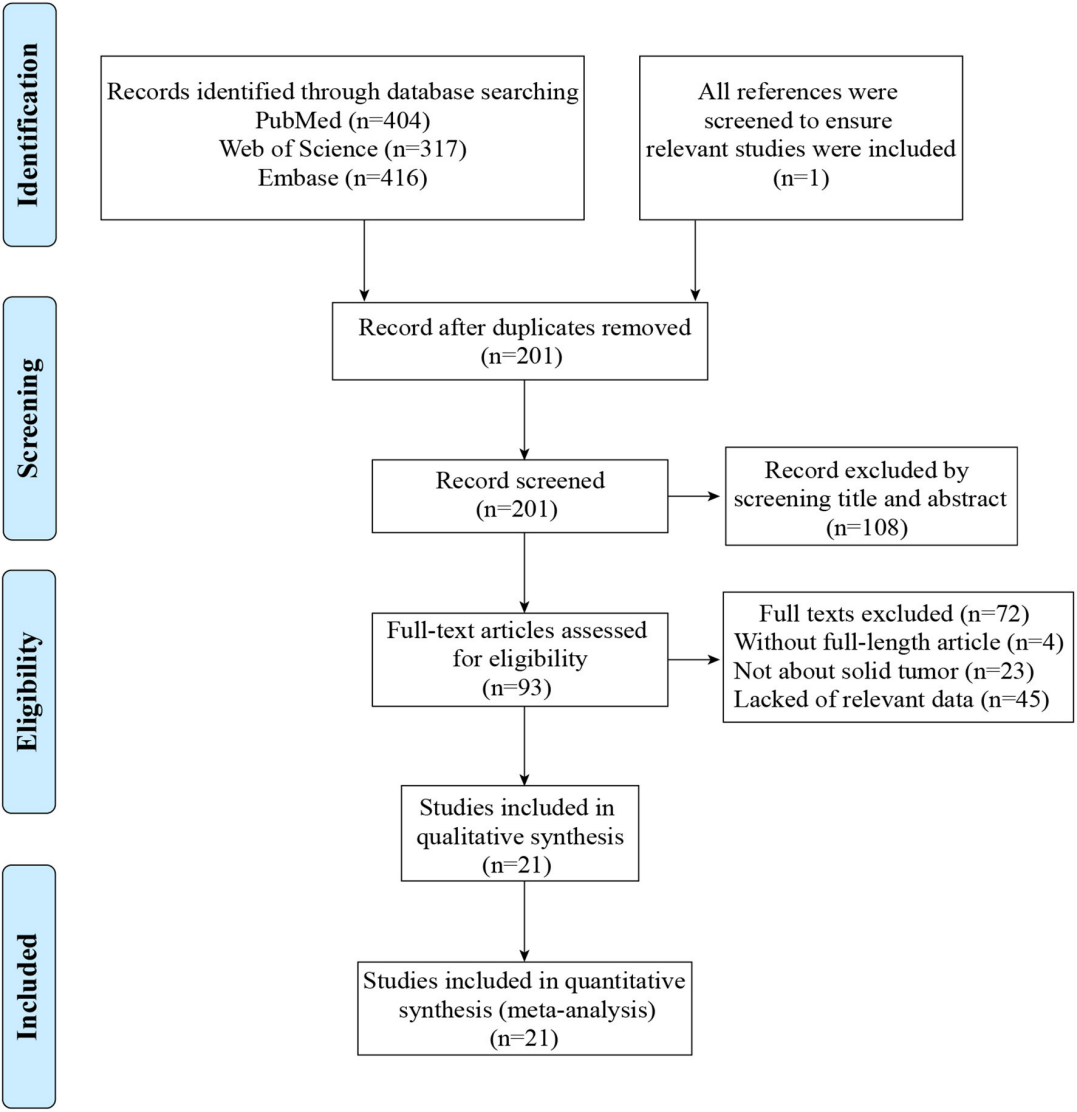
Flow diagram for the process of studies selection.

A flow diagram showing the study selection was presented in Figure 1. A total of 1,138 articles were obtained by using the described searching strategy mentioned above. 937 articles were excluded on account of duplication. The remaining 201 records were screened on the base of title, abstract, and full-text. Finally, we retained 21 manuscripts to investigate the correlation between TIM-3 expression and patient survival in various solid malignant tumors (9–14, 28–42).

### Study Characteristics

A total of 3,072 patients, ranging from 30 to 587 patients per study, were involved in our meta-analysis. Among all eligible studies, 13 studies focused on the TIM-3 expression on tumor cells (10–14, 28–33, 36, 39), six studies centered on the TIM-3 expression on TILs (9, 34, 35, 37, 38, 40), one study combined the TIM-3 expression on tumor cells and TILs (41), and one study analyzed the TIM-3 expression on tumor cells and TILs separately (42). All researches utilized the IHC techniques to detect the expression level of TIM-3. The studies were published from 2012 to 2019 with the patients from China (*n* = 17), Japan (*n* = 1), Korea (*n* = 2), and Poland (*n* = 1). The type of carcinoma included HCC, CRC, BUC, esophageal squamous cell carcinoma (ESCC), NSCLC, GC, RCC, cervical cancer, breast cancer, pancreatic cancer, prostate cancer, osteosarcoma, and skull base chordoma. The cut-off value of each study was not exactly the same. Among all included studies, eight studies reported the HRs and 95% CIs directly. In the remaining 13 studies, HRs and 95% CIs were estimated from survival curves. OS was reported in all studies, while DFS and PFS were addressed in seven and one study, respectively. Additionally, 13 studies showed the association between TIM-3 and clinical-pathological characteristics. The details regarding the characteristics of the 21 eligible studies were listed in Table 1.

**Table 1:** Main characteristics of the studies included in the meta-analysis.

**First author**	**Year**	**Patient source**	**Sample size**	**Cancer type**	**Method**	**Expression location**	**Cut-off value**	**Median(range) months**	**Outcome**	**M/U**	**HR ratio**	**NOS**
Li et al. (10)	2012	Chinese	99	HCC	IHC	Tumor cell	Median level	NA	OS	U	Survival curve	6
Zhuang et al. (11)	2012	Chinese	30	NSCLC	IHC	Tumor cell	≥25% positivity cell	34 (1-78)	OS	M	Reported	9
Jiang et al. (28)	2013	Chinese	305	GC	IHC	Tumor cell	HSCORE>0	40 (3-135)	OS	U	Survival curve	8
Cao et al. (29)	2013	Chinese	43	Cervical cancer	IHC	Tumor cell	Score≥2	45.2 (5-60)	OS	U	Survival curve	6
Yang et al. (13)	2015	Chinese	100	BUC	IHC	Tumor cell	HSCORE≥100	44 (3-60)	DFS	U	Survival curve	8
Yang et al. (13)	2015	Chinese	100	BUC	IHC	Tumor cell	HSCORE≥100	44 (3-60)	OS	U	Survival curve	8
Zhou et al. (30)	2015	Chinese	201	CRC	IHC	Tumor cell	HSCORE≥200	61 (2-103)	OS	U	Survival curve	8
Komohara et al. (12)	2015	Japanese	91	RCC	IHC	Tumor cell	Score≥1	NA	OS	U	Survival curve	6
Komohara et al. (12)	2015	Japanese	91	RCC	IHC	Tumor cell	Score≥1	NA	PFS	U	Survival curve	6
Shan et al. (31)	2016	Chinese	64	ESCC	IHC	Tumor cell	Score>3	31 (7-105)	OS	U	Survival curve	8
Hou et al. (32)	2017	Chinese	45	ESCC	IHC	Tumor cell	Score≥3	NA	OS	M	Reported	6
Peng et al. (33)	2017	Chinese	50	Pancreatic cancer	IHC	Tumor cell	Score≥3	10.3	OS	U	Survival curve	7
Wu et al. (14)	2017	Chinese	139	Prostate cancer	IHC	Tumor cell	Score≥6	22.1	OS	U	Survival curve	7
Byun et al. (34)	2018	Korea	109	Breast cancer	IHC	TIL	Score≥2	76 (6-131)	DFS	M	Reported	8
Byun et al. (34)	2018	Korea	109	Breast cancer	IHC	TIL	Score≥2	76 (6-131)	OS	M	Reported	8
Duan et al. (35)	2018	Chinese	95	ESCC	IHC	TIL	≥1% positivity cell	32 (3-84)	OS	U	Survival curve	7
Wang et al. (9)	2018	Chinese	587	GC	IHC	TIL	Median	48 (1-117)	OS	M	Reported	8
Su et al. (41)	2018	Chinese	223	NSCLC	IHC	Tumor cell +TIL	≥24% on tumor cell and/or≥11% on TIL	76 (4-101)	DFS	M	Reported	8
Su et al. (41)	2018	Chinese	223	NSCLC	IHC	Tumor cell +TIL	≥24% on tumor cell and/or≥11% on TIL	76 (4-101)	OS	M	Reported	8
Cheng et al. (36)	2018	Chinese	42	Breast cancer	IHC	Tumor cell	Score>1	NA	OS	U	Survival curve	8
Jia et al. (42)	2019	Poland	139	NSCLC	IHC	Tumor cell	>5% on tumor cell	NA	DFS	U	Survival curve	7
Jia et al. (42)	2019	Poland	139	NSCLC	IHC	TIL	>10% on TIL	NA	DFS	U	Survival curve	7
Jia et al. (42)	2019	Poland	139	NSCLC	IHC	Tumor cell	>5% on tumor cell	NA	OS	U	Survival curve	7
Jia et al. (42)	2019	Poland	139	NSCLC	IHC	TIL	>10% on TIL	NA	OS	U	Survival curve	7
Zhao et al. (37)	2019	Chinese	183	ESCC	IHC	TIL	Score>3	NA	DFS	M	Reported	8
Zhao et al. (37)	2019	Chinese	183	ESCC	IHC	TIL	Score>3	NA	OS	M	Reported	8
Hong et al. (38)	2019	Korea	396	ESCC	IHC	TIL	≥1%	24.8 (0.5–210)	DFS	M	Reported	9
Hong et al. (38)	2019	Korea	396	ESCC	IHC	TIL	≥1%	24.8 (0.5–210)	OS	M	Reported	9
Pu et al. (39)	2019	Chinese	38	Osteosarcoma	IHC	Tumor cell	Score >1.1	NA	OS	U	Survival curve	8
Zhou et al. (40)	2019	Chinese	93	Skull base chordoma	IHC	TIL	Score>1	36.9 (14-66)	DFS	M	Reported	9
Zhou et al. (40)	2019	Chinese	93	Skull base chordoma	IHC	TIL	Score≥2	36.9 (14-66)	OS	M	Reported	9

*HCC, hepatocellular carcinoma; NSCLC, non-small cell lung cancer; GC, gastric cancer; BUC, bladder urothelial carcinoma; CRC: colorectal cancer; RCC: renal cell carcinoma; ESCC, esophageal squamous cell carcinoma; IHC, Immunohistochemistry; TIL, tumor infiltrating lymphocyte; DFS, disease-free survival; PFS, progression-free survival; OS, overall survival; M, multivariate analysis; U, univariate analysis; HR, hazard ratio; NA, not available; NOS: Newcastle-Ottawa Scale*.

### Association of TIM-3 Expression With Overall Survival

**Figure 2:**
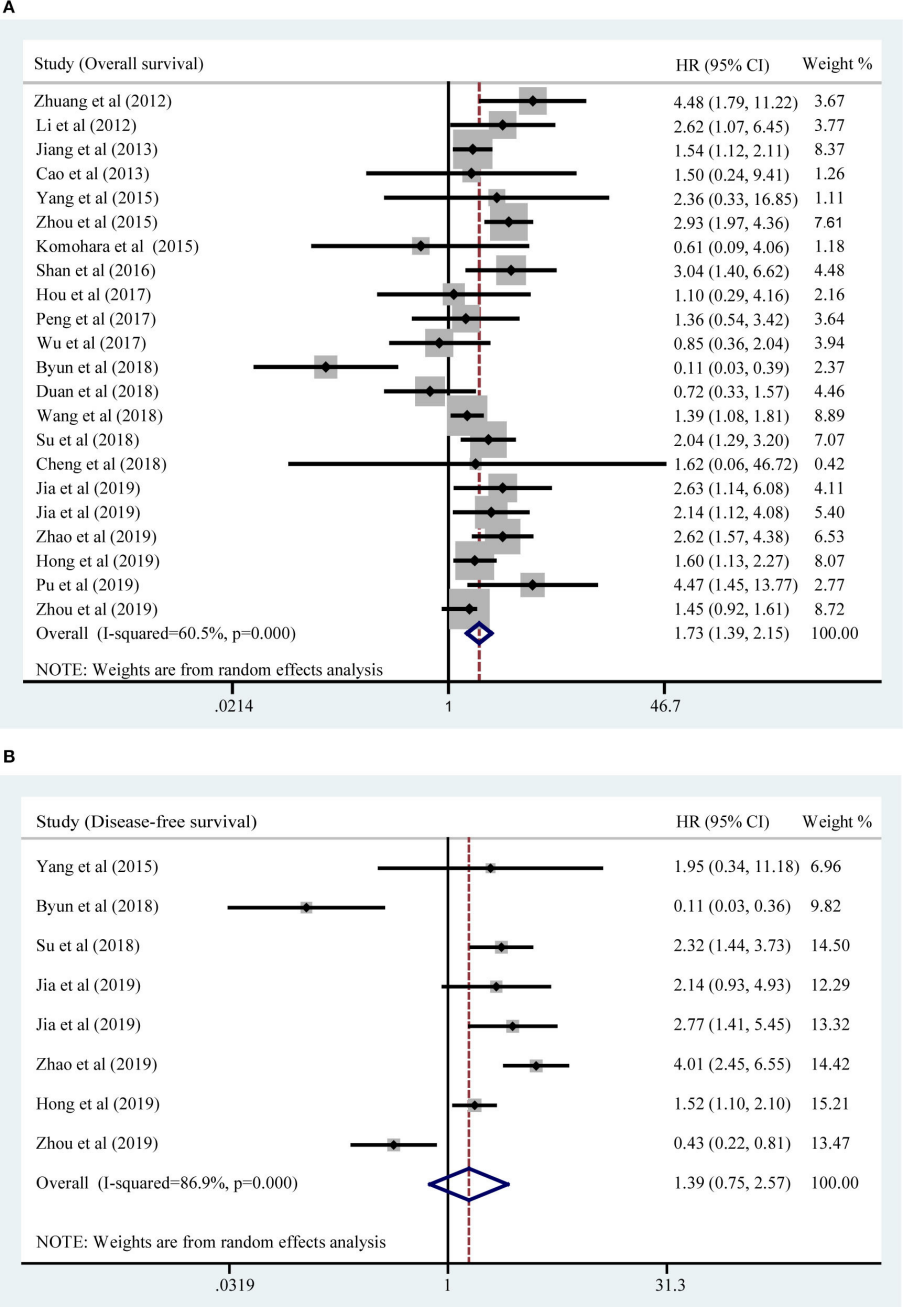
Forest plot of HR for the association of TIM-3 overexpression and OS **(A)** and DFS **(B)**.

**Figure 3:**
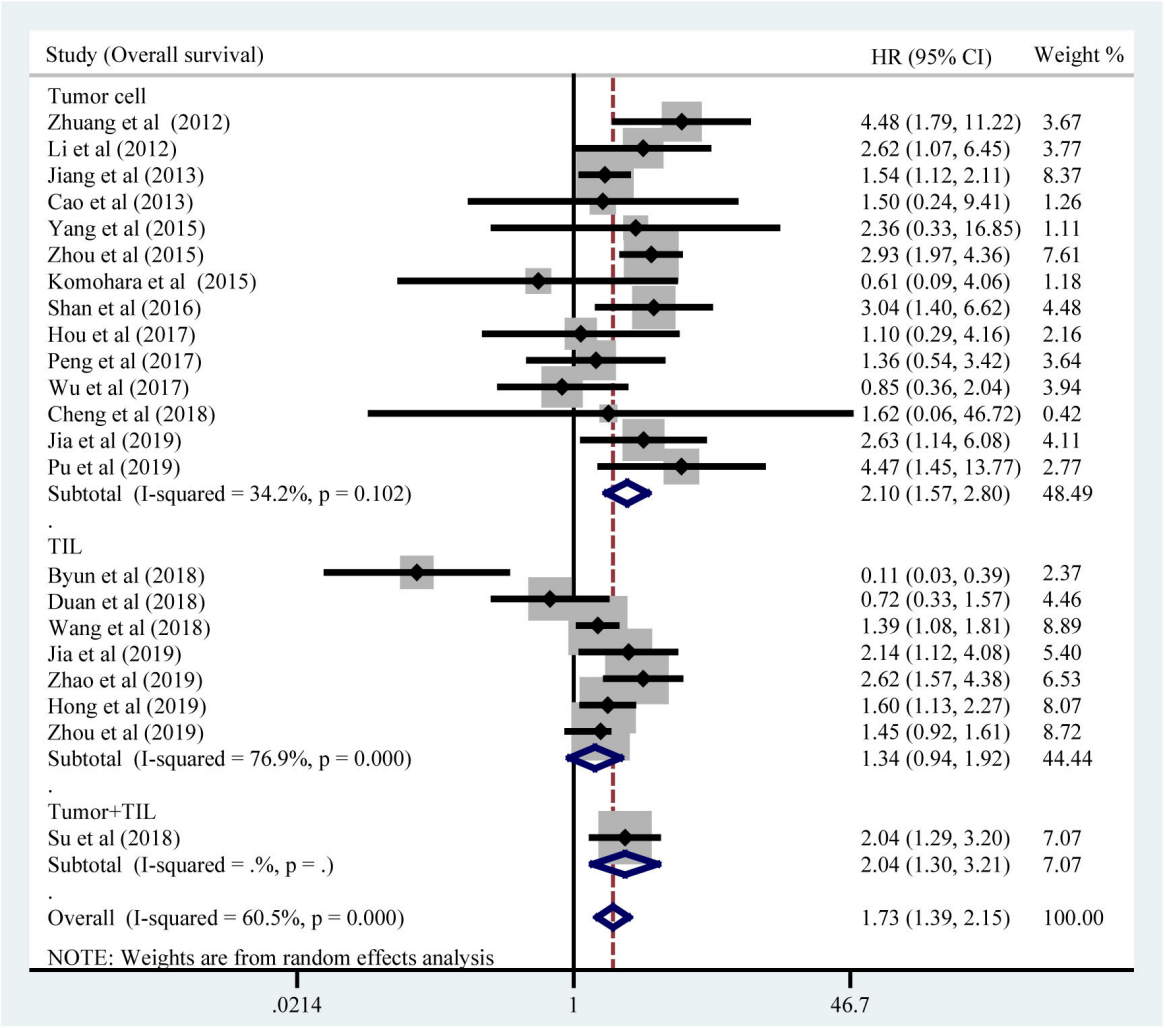
Subgroup analysis of the association betweenTIM-3 and OS in tumor cells and TILs.

All included studies supplied suitable data to analyze the association between TIM-3 on tumor cells/TILs and OS. The main results of this meta-analysis were listed in Table 2. As the heterogeneity test reported a P_H_ <0.001 and *I*^2^ value of 60.5%, we used a random effects model to pool the HR. As shown in Figure 2A, TIM-3 upregulation was significantly associated with a worse OS in patients with solid cancer (HR = 1.73; 95% CI = 1.39–2.15; *P* < 0.001). In order to seek the source of inter-study heterogeneity, subgroup analysis was further performed based on the expression position, cancer type, sample size, and method to estimate HR (Table 2). Overexpressions of TIM-3 on tumor cells were significantly related to unfavorable OS (HR = 2.10; 95% CI = 1.57–2.80; *P* < 0.001) (Figure 3). The TIM-3 expression on TILs showed a tendency of increased risk of short OS, however, the association did not research a statistical difference (HR = 1.34; 95% CI = 0.94–1.92; *P* = 0.105). Elevated TIM-3 as a negative predictor on OS was confirmed in patients with ESCC (HR = 1.70; 95% CI = 1.08–2.70; *P* = 0.023), NSCLC (HR = 2.35; 95% CI = 1.71–3.24; *P* < 0.001), GC (HR = 1.45; 95% CI = 1.19–1.77; *P* < 0.001) and other types of cancers (HR = 1.83; 95% CI = 1.25–2.68; *P* = 0.002), but not in patients with breast cancer (HR = 0.27; 95% CI = 0.02–3.10; *P* = 0.292).

**Table 2:** Pooled HRs for OS and subgroup analysis of TIM-3 expression in solid cancer patients.

**Categories**	**Number of studies**	**Number of patients**	**Random effects model**		**Heterogeneity**		***P*-value**
			**Pooled HR**	**95% CI**	***I*^**2**^**	**P_H_ value**	
OS	21	3,072	1.73	1.39–2.15	60.5%	<0.001	<0.001
**EXPRESSION POSITION**							
Tumor cells	14	1,386	2.10	1.57–2.80	34.2%	0.102	<0.001
TILs	7	1,602	1.34	0.94–1.92	76.9%	<0.001	0.105
Tumor cells+TILs	1	223	2.04	1.29–3.20	-	-	0.002
**CANCER TYPE**							
ESCC	5	783	1.70	1.08–2.70	60.1%	0.040	0.023
NSCLC	3	392	2.35	1.71–3.24	0.0%	0.489	<0.001
GC	2	892	1.45	1.19–1.77	0.0%	0.635	<0.001
Breast cancer	2	151	0.27	0.02–3.10	53.6%	0.142	0.292
Others	9	854	1.83	1.25–2.68	49.4%	0.045	0.002
**SAMPLE SIZE**							
<100	11	690	1.81	1.24–2.64	43.4%	0.061	0.002
≥100	10	2,382	1.67	1.25–2.24	71.8%	<0.001	0.001
**METHOD TO ESTIMATE HR**							
Multivariate	8	1,666	1.57	1.12–2.22	75.4%	<0.001	0.01
Univariate	13	1,406	1.87	1.40–2.50	41.8%	0.051	<0.001

*OS, overall survival; TIL, tumor infiltrating lymphocyte, ESCC, esophageal squamous cell carcinoma; NSCLC: Non-small cell lung cancer; GC, gastric cancer*.

### Association of TIM-3 Expression With DFS

Seven studies comprising 1,243 patients assessed the link between TIM-3 and DFS in cancer patients, among which one study presented the data about the TIM-3 on tumor cells and TILs separately. A random effects model was used on account of the obvious heterogeneity among these studies (*I*^2^ = 86.9%, P_H_ <0.001). The result showed that TIM-3 expression was not associated with DFS (HR = 1.39; 95% CI = 0.75–2.57; *P* = 0.297) (Figure 2B). Subsequently, subgroup analysis was performed (Table 3). We observed that elevated TIM-3 expression was correlated significantly with short DFS in NSCLC patients (HR = 2.40; 95% CI = 1.69–3.41; *P* < 0.001) and high level of TIM-3 significantly correlated with short DFS in study where HR was extrapolated by univariate analysis (HR = 2.45; 95% CI = 1.48–4.05; *P* < 0.001). As PFS was reported in only one related study, it was insufficient to conduct a meta-analysis.

**Table 3:** Pooled HRs for DFS and subgroup analysis of TIM-3 expression in solid cancer patients.

**Categories**	**Number of studies**	**Number of patients**	**Random effects model**		**Heterogeneity**		***P*-value**
			**Pooled HR**	**95% CI**	***I*^**2**^**	**P_H_ value**	
DFS	7	1243	1.39	0.75–2.57	86.9%	<0.001	0.297
**EXPRESSION LOCATION**							
Tumor cells	2	239	2.10	0.99–4.46	0.0%	0.925	0.053
TILs	5	920	1.06	0.42–2.65	92.1%	<0.001	0.904
Tumor cell+TILs	1	223	2.32	1.44–3.73	-	-	0.001
**TUMOR TYPE**							
ESCC	2	579	2.42	0.94–6.26	90.4%	0.001	0.068
NSCLC	2	362	2.40	1.69–3.41	0.0%	0.876	<0.001
Others	3	302	0.40	0.11–1.46	73.9%	0.022	0.164
**SAMPLE SIZE**							
<100	1	93	0.43	0.22–0.82	-	-	0.010
≥100	6	1,150	1.71	0.97–3.04	82.4%	<0.001	0.066
**METHOD TO ESTIMATE HR**							
Multivariate	5	1,004	1.05	0.45–2.44	92.1%	<0.001	0.916
Univariate	2	239	2.45	1.48–4.05	0.0%	0.864	<0.001

*DFS, disease-free survival; TIL, tumor infiltrating lymphocyte, ESCC, esophageal squamous cell carcinoma; NSCLC: Non-small cell lung cancer*.

### Association of TIM-3 With Clinicopathological Parameters

Relevant data were utilized to extrapolate the relationship between TIM-3 and ten clinical-pathological features. These parameters included gender, age, T stage, lymph node metastasis, tumor grade, TNM stage, smoking history, distant metastasis, vascular invasion, and PD-1 expression. The overall results were showed in Table 4 in detail. The synthesized data indicated that TIM-3 expression had no obvious association with patients' sex, age, T stage, smoking history, or vascular invasion. However, significant connections were presented between TIM-3 and lymph node metastasis (OR = 1.59; 95% CI = 1.10–2.29; *P* = 0.013), tumor grade (OR = 1.68; 95% CI = 1.21–2.34; *P* = 0.002), as well as PD-1 expression (OR = 3.26; 95% CI = 2.20–4.82; *P* < 0.001).

**Table 4:** Association between TIM-3 and clinical parameters in solid cancers.

**Categories**	**Number of studies**	**Model**	**OR (95% CI)**	**Heterogeneity**		***P*-value**
				***I*^**2**^**	**P_H_ value**	
Gender (Female vs. Male)	13	Fixed	1.01 (0.83–1.24)	0.0%	0.966	0.893
Age (≥60 vs. <60)	12	Fixed	1.19 (0.99–1.43)	0.0%	0.601	0.064
T stage (T3~T4 vs. T1~T2)	8	Random	1.07 (0.59–1.95)	79.2%	<0.001	0.825
Lymph node metastasis (N+ vs. N–)	8	Random	1.59 (1.10–2.29)	56.1%	0.025	0.013
Grade (Grade2–3 vs. Grade1)	9	Fixed	1.68 (1.21–2.34)	0.0%	0.700	0.002
TNM Stage (III–IV vs. I–II)	12	Random	1.41 (0.91–2.17)	69.3%	<0.001	0.122
Smoking (Yes vs. no)	6	Fixed	1.13 (0.85–1.50)	39.4%	0.129	0.394
Distant metastasis (Yes vs. no)	2	Fixed	1.58 (0.58–4.33)	42.3%	0.177	0.371
Vascular invasion (Yes vs. no)	4	Random	1.22 (0.71–2.12)	57.6%	0.070	0.471
PD−1(High vs. low)	3	Fixed	3.26 (2.20–4.82)	40.8%	0.185	<0.001

*N-, lymph node negative; N+, lymph node positive; PD-1; programmed cell death protein-1*.

### Sensitivity Analysis

**Figure 4:**
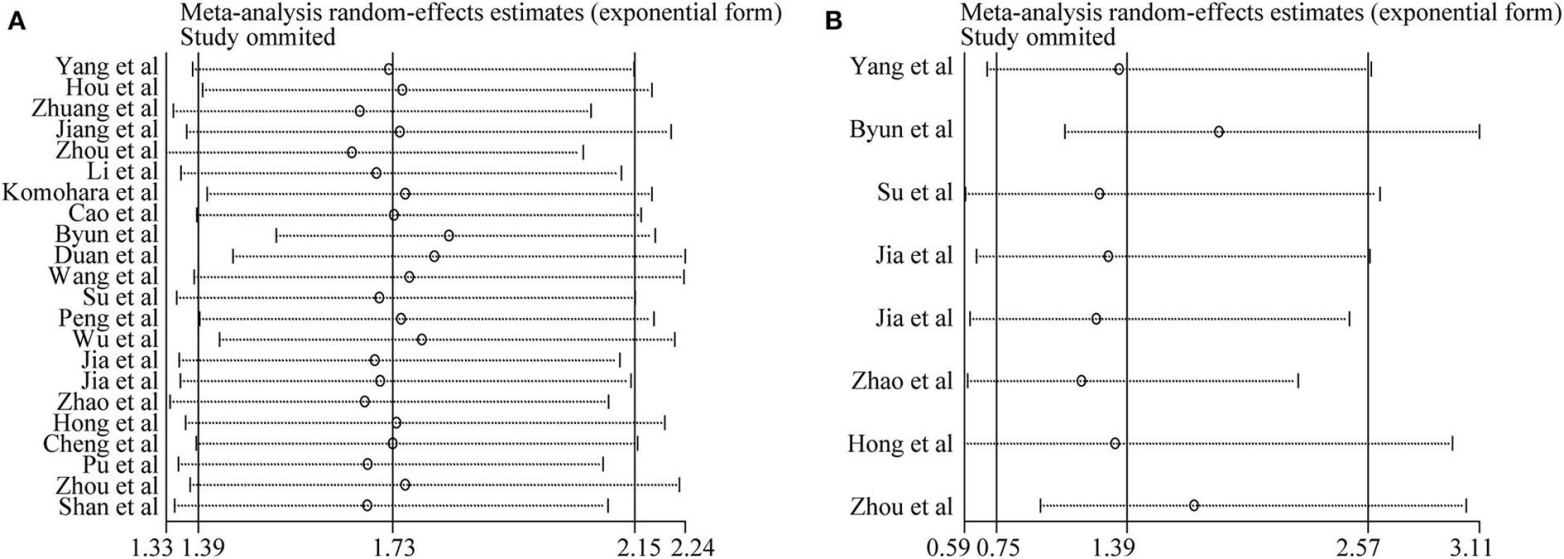
Sensitivity analysis of pooled HRs on the association between TIM-3 expression and OS **(A)** as well as DFS **(B)**.

Sensitivity analysis was conducted to assess the influence of each study on the synthetic results of meta-analysis by omitting one study at a time. As shown in Figures 4A,B, there was no significant change after omitting any single study for the effect of TIM-3 expression on OS or DFS, indicating the stability of our meta-analysis.

### Publication Bias

**Figure 5:**
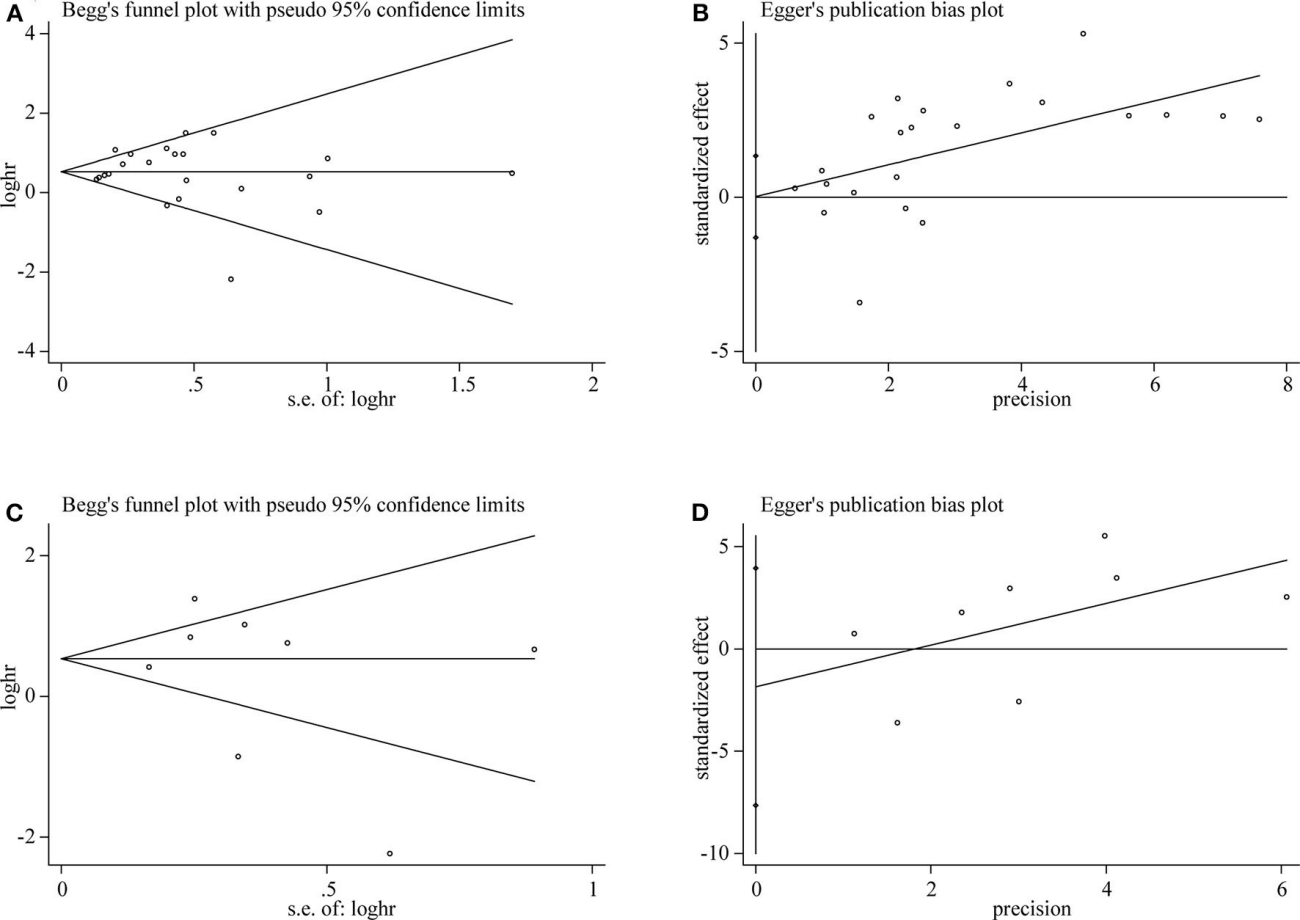
Publication bias detected by Begg's test and Egger's test. Begg's **(A)** and Egger's **(B)** test for OS. Begg's **(C)** and Egger's **(D)** test for DFS.

Both Begg's test and Egger's test were used to evaluate publication bias of OS and DFS analysis. The shape of funnel plot did not appear dissymmetric, and Egger's test also showed no publication bias among the studies analyzing the association of TIM-3 expression and OS (Figures 5A,B). No significant asymmetry was observed in the funnel plot for the relation of TIM-3 expression with DFS (*P* = 0.536, Figure 5C). Moreover, the conclusion was confirmed by Egger's tests (*P* = 0.465, Figure 5D). Hence, publication bias was not presented in our meta-analysis.

### Expression of TIM-3 mRNA and Prognosis in Database Test

**Figure 6:**
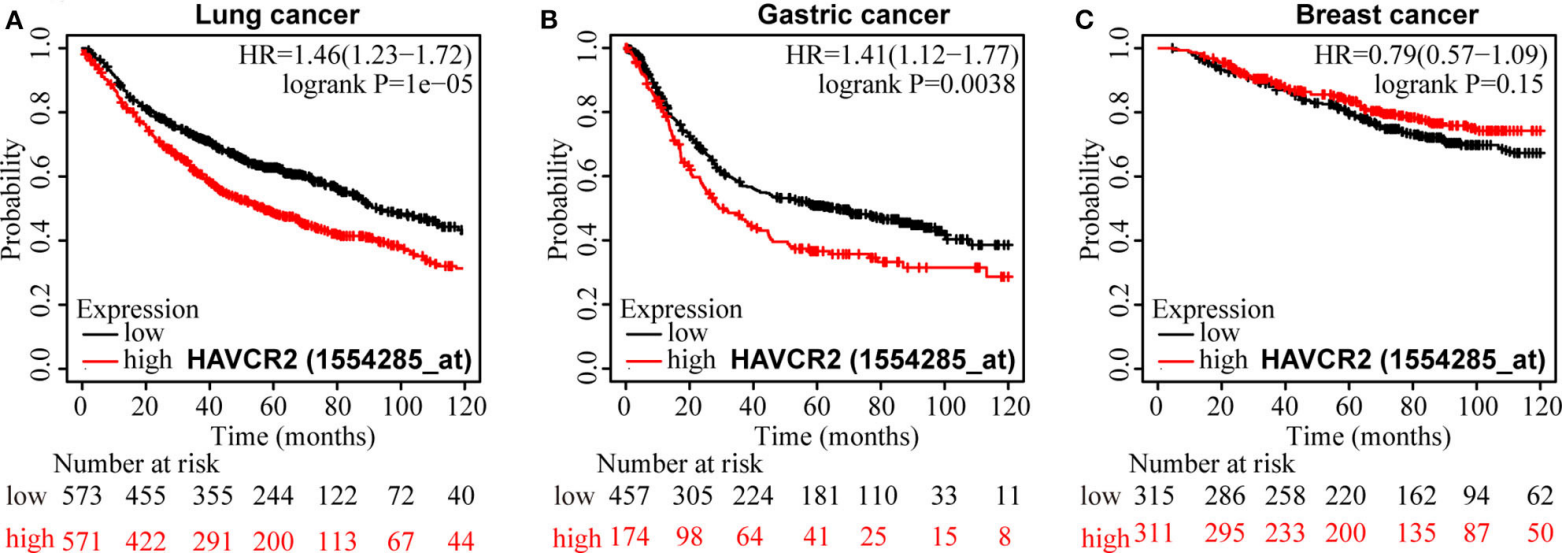
Kaplan-Meier survival curves for OS according to TIM-3 mRNA expression in patients with NSCLC **(A)**, GC **(B)**, and breast cancer **(C)**.

For OS, a significant correlation was revealed between high TIM-3 mRNA expression and poor OS in patients with NSCLC (HR = 1.46, *P* < 0.001) (Figure 6A) and GC (HR = 1.41, *P* = 0.0038) (Figure 6B), but not in patients with breast cancer (HR = 0.79, *P* = 0.51) (Figure 6C). The results of TIM-3 mRNA expression adopted from public database were in accordance with those of our combined result of subgroup analysis of TIM-3 protein abundance based on cancer type.

## Discussion

The identification of TIM gene family was found in searching for asthma susceptibility genes (4). TIM genes family is located on human chromosome 5q33—a region which has been repeatedly linked to allergic and autoimmune diseases (43–45). TIM-3 blockade exacerbated the disease phenotype in experimental allergic encephalomyelitis animal models, indicating TIM-3 as a negative regulatory molecule (3). TIM-3 is widely expressed on immune cells, such as T cells, NK cells (46), DCs and monocytes/macrophages (47, 48). The results of Yan et al. strongly suggested that increased TIM-3 expression in monocytes/tumor-associated macrophages was positively correlated with higher tumor grades and the poor survival in patients with HCC (48). In the most recent, TIM-3 expression had also been reported on malignant tumor cells, such as melanoma (49), CRC (8), GC (9), and HCC (10). Ma's group thoroughly delineated that tumor cell-intrinsic TIM-3 exerted protumoral activity via NF-κB/IL-6/STAT3 axis (50). Xiao et al. demonstrated that intrinsic expression of TIM-3 in nasopharyngeal carcinoma (NPC) cells promoted epithelial-mesenchymal transition of NPC through SMAD7/SMAD2/SNAIL1 axis (51). In these cancers, the prognostic significance of TIM-3 was evaluated, but there was no consistence on the relationship between the expression of TIM-3 detected by IHC and survival in patients.

Our meta-analysis, including 3,072 cases from 21 published studies, calculated a combined HR of 1.73 (95% CI = 1.39–2.15; *P* < 0.001), which supported that high TIM-3 expression was correlated with poor OS. However, a significant heterogeneity in OS across the included studies was existed. Thus, we performed sensitivity analysis to ascertain the cause for heterogeneity. The result suggested that pooled HR was not clearly influenced by any single study. We further performed subgroup analysis. Interestingly, subgroup analysis showed that TIM-3 overexpression on tumor cells was associated with poor OS, but the association between TIM-3 expression on TILs and OS didn't reach a statistical significance. TIM-3 was mainly expressed on immune cells with lower expression in tumor cells (9, 42). It seems that the expression of TIM-3 on tumor cells has greater prognosis value, but the exact mechanism needs further investigation. In the subgroup analysis based on cancer types, TIM-3 showed the inconsistent prognostic effects. Elevated TIM-3 expression was connected with poor OS in ESCC, NSCLC, GC, and other cancers, but was not in breast cancer. We noticed that one study conducted by Byun et al. (34) selected a special type of breast cancer—triple-negative breast cancer. This study indicated that TIM-3 expression on TILs was a positive prognosticator. So, lack of consistency of these studies may be due to the different features of various tumors and the distinct expression location.

Furthermore, we adopted Kaplan-Meier plotter to explore the prognostic value of TIM-3 mRNA in lung cancer, GC, and breast cancer with the public database from GEO and TCGA to verify our finding. It demonstrated that the OS of lung cancer and GC patients with high TIM-3 was extremely poor, which was in accordance with our pooled HR results, suggesting that TIM-3 mRNA expression reflected protein abundance. Besides, for DFS, elevated expression of TIM-3 didn't affect prognosis. But in subgroup of NSCLC and univariate analysis, TIM-3 was found to be correlated with poor DFS. As the studies involved in the analysis of DFS were relative deficiency, the conclusion was not convincing. As only one study reported the relationship between PFS and TIM-3, we could not calculate the pooled HR. Moreover, we stratified the variables by clinicopathological features, a higher level of TIM-3 showed a significant correlation with poor differentiation, lymph node metastasis, and higher PD-1 expression.

PD-1(CD279), an immune checkpoint molecule belonging to the CD28 superfamily, suppresses the activation and function of T cells by binding with its ligands PD-L1 and PD-L2 (52, 53). Immune checkpoint blockade therapy targeted PD-1/PD-L1 was one of the most well-established immunotherapies (54). Nevertheless, drug resistance remains a daunting challenge (55). Now, TIM-3 is classified as negative immune checkpoint molecule similar to PD-1. Studies have shown that the majority of TIM-3+TILs co-expressed PD-1, indicating a potential synergistic effect between these two negative immune checkpoints (10, 56). TIM-3+PD-1+TILs reflected a more exhausted phenotype as defined by failure to proliferate and secret less IFN-γ, IL-2 and tumor necrosis factor-α (57, 58). Encouragingly, the evidence from preclinical studies showed that dual blockade of TIM-3 and PD-1 pathway effectively restricted tumor growth (59). Nowadays, several anti-TIM-3 mAbs were evaluated in clinical trials as a monotherapy or in combination with PD-1/PD-L1 mAbs (18, 60).

A meta-analysis of the association between TIM-3 expression and cancer prognosis had been previously reported by Zhang et al. (61). They collected seven studies in seven different cancer types that involved 869 patients and the result indicated that high TIM-3 expression was significantly correlated to poor OS. Additionally, higher TIM-3 expression was associated with advanced tumor stage in their analysis (61). Compared with the previous study, our meta-analysis has several advantages as well as limitations. Our meta-analysis reviewed the role of TIM-3 in both OS and DFS in more cancer types. Besides, we conducted the subgroup analysis which was not performed before. Furthermore, to ensure the credibility of results, we have expanded the number of studies for analysis.

Although we have made every effort to conduct a comprehensive analysis, some limitations still exist in our meta-analysis inevitably. Firstly, most of the subjects were from East Asia, which could have resulted in selection bias. Thus, the conclusion should be reassessed in European patients. Secondly, the number of eligible studies included in PFS and DFS were relatively small, which may cause heterogeneity. More studies are needed to explore the relationship among TIM-3, DFS, and PFS. Thirdly, although all studies employed IHC, the antibodies they used were not exactly the same, and the threshold value was inconsistent among trials, which may contribute to the observed heterogeneity. Establishment of the unified cut-off value should be further explored. Finally, some studies didn't provide the HRs directly, so we extracted HRs and 95% CIs based on survival curves, which may influence the accuracy of data.

## Conclusion

In summary, our meta-analysis illustrated that TIM-3 expressions on tumor cells were significantly associated with poor OS but not DFS in most human solid cancers. TIM-3 expression was also positively connected with lymph node metastasis, tumor grade, and PD-1 expression. It seems that TIM-3 is not merely an indicator of tumor prognosis but also a promising therapeutic target for solid tumors. Due to the inevitable limitations, our results should be interpreted with caution and further prospective multicenter studies with larger sample size will be necessary to determine the role of TIM-3 in both the prognostic prediction and targeted therapy for various types of cancers.

## Data Availability Statement

All datasets presented in this study are included in the article/supplementary material.

## Author Contributions

SQ analyzed the data and wrote the manuscript. SQ and BD searched and collected the literatures. MY and QC contributed to statistical analysis. KW designed the study and revised the manuscript. All authors participated in the discussion, read, and approved the final manuscript.

## Conflict of Interest

The authors declare that the research was conducted in the absence of any commercial or financial relationships that could be construed as a potential conflict of interest.
